# Post-Graduate Study in Europe

**Published:** 1908-09

**Authors:** Otis H. Johnson

**Affiliations:** Athens, Ga.


### POST-GRADUATE STUDY IN EUROPE.

### WITH SPECIAL REFERENCE TO EYE, EAR, NOSE AND THROAT.

OTIS H. JOHNSON, B. A., M. D., ATHENS, GA.

Post-graduate study in the various medical centers of Europe has long had ardent advocates among American physicians, but it has been only in recent years that they have in any great numbers forsaken their own great cities for foreign study. The change has been brought about by a number of causes, probably the greatest of which is the tendency of the profession to specialize, along all lines, the inadequacy of American courses and scarcity of practical teaching in special branches, In our post-graduate schools and hospitals the specializing physicion, who is unwilling or unable to take service upon the house staff of some special hospital, can usually obtain no teaching and practice beyond that prepared for the general practitioner by the post-graduate schools, and special hospitals pay little or no attention to clinical and practical teaching. A number of such special institutions advertise "unexcelled clinical advantages and teaching," but the student generally finds that after paying his fee in the office he receives no more attention, and is looked upon rather as an intruder. The advantages are here most certainly, but they are not for the student, and the teaching is conspicuous only by its absence.

Of course it is possible for some to find clinics in which minor positions upon the attending staff may be obtained for the asking or for money, but only a favored few have this good fortune, and it may take months of search and waiting to find a satisfactory appointment.

Heretofore many Americans have been unwilling to go to Germany and Austria for special work, because they considered a knowledge of the German language essential, but of late years they have learned that courses on any subject in the medical line can be obtained in English in Vienna, and to a lesser degree in Berlin. Not a word of German need be learned for the successful prosecution of a course of study in Vienna.

Again, the sharp reduction in rates caused by the struggle for patronage between the English and the German transatlantic steamship companies has drawn an unprecedent number of Americans to Europe within the past year. In Vienna alone, in November, 1907, there were over two hundred American physicians, and other medical centers had correspondingly large delegations.

The advantages of European study are many. Granted, for the sake of argument, that its advantages are only equal to those offered by our own hospitals, the prestige alone of work abroad is well worth the trip. But, aside from this rather unworthy consideration, European hospitals actually offer more in the same length of time, more systematized work, more thorough work for the student, and at less cost, than American hospitals.

A great objection to practical work in our hospitals is that no clinics whatever are held in the morning, except those in the regular post-graduate schools, and the practitioner, who is generally at heavy expense both at home and at his place of study, is obliged to waste half a day every day before getting any work. Of course this may not apply in every city, nor to every specialty, but it is certainly the general rule. The American physician in a large city must have his office hours in the morning, and therefore cannot hold clinics during that time. In Europe the physician has his office hours at a time which will not interfere with his clinic, and consequently a student can attend clinics there from eight in the morning until eight or nine at night. The amount of experience to be gained is limited only by one's endurance.

London offers splendid opportunities for general work, diseases of children, skin, G. U., opsonic work, and eye. The skin clinics there are immense, and it is probably the best place in the world to study this specialty. Genito-urinary work is also excellent, and there are unusual and numerous opportunities for observing prostatectomies. There is good clinical and practical instruction in diseases of children here, and the clinics are large. For the embryo oculist, who has done no previous work in this line, Moorefield Eye Hospital (or Royal Ophthalmic Institute), London, offers splendid advantages. Every student who is enrolled here is called a "Junior Assistant," and, after five month's work is given a certificate stating that he has served a term as such, and has enjoyed the advantages of the "practice of the Hospital." Systematic courses are given here four times a year, beginning on the first of January, April, July, and October, upon refraction, operative work on pigs' eyes and the cadaver, external diseases, examination, muscles, pathology, ophalmoscopy, etc. These courses contain from four to twelve lectures or lessons each, and are spread over the term of three months. Those on pathology under the famous English pathologist, Parsons, and on ophthalmoscopy, illustrated and demonstrated by over a hundred patients with pathological fundi for the student to examine, are especially good.

The student at Moorefield signs up with two of the chief surgeons, reports each morning for duty from nine to one, is given clinical demonstrations by the surgeon in charge, refracts, examines patients, perhaps does a minor operation or two, and studies what pathological fundi there may be encountered in the day's clinic. He does not live in the Hospital, nor does he wear a uniform, and he is free in the afternoon to attend clinics in other hospitals. Ear, nose, and throat courses are not satisfactory in London, and those offered at Gray's Inn Hospital and at Golden Square Hospital, which are the best, are too elementary for anyone save a novice.

Paris offers no inducements for post-graduate work, and is generally passed up by the American student, except when on pleasure bent.

Berlin has been the favorite medical center in days gone by, when only German-speaking Americans studied there, but it has yielded first place to Vienna since the surgeons of the latter city have made a specialty of giving courses in English. In Berlin an American is seriously handicapped in his studies if he does not speak German, and it is often necessary to spend several months studying the language there before he can make any progress at all in his work, although some of the courses are given in English. There are many hospitals here, and they are situated in different parts of the city, necessitating quite a loss of time to the student who cannot arrange for all his work in one hospital.

The Anglo-American Club of Berlin will be found of great assistance to the American stranger.

Vienna should be the Mecca of all Americans seeking post-graduate medical work, for here the student can get courses in English upon any subject, under world-famous men, and the work is all concentrated in the immense "Allgemeine Krankenhaus," containing three thousand beds, and the smaller "Poliklinik," only a short block distant. Here the surgeons in charge of the clinics make a specialty of speaking English and giving post-graduate instruction to Americans, for doing which they have been accused by their brethren in Berlin and London, of "selling themselves to the Americans."

The courses here are given under the supervision of the American Medical Association of Vienna, which looks after newcomers, helps them to begin their work, to find rooms and pensions, regulates as far as possible the number of students in each course, and by co-operation with the professors assures each one fair play. This excellent organization holds regular, well-attended meetings in an amphitheater of the "Allgemeine Krankenhaus," and publishes, for the benefit of the strangers, a complete list of all courses usually taken by Americans, announcing the time, place, fee, duration and number of places of each. Also, by its efforts these courses have become affiliated with the University of Vienna, and after taking a certain number of them during a stay of not less than three months, a post-graduate certificate is issued by the University, if so desired.

Vienna courses generally last about twenty hours, one hour each day except Sunday, though some are shorter, and some longer. They are usually limited to eight or ten men, and are repeated as often as the minimum number signs up on the list, which is posted on the notice board of the American Medical Association, at its headquarters in Cafe Klinik. During the Winter the most popular courses are often signed up for six or eight weeks in advance, but others on the same subject are always being arranged, and a new comer can rely upon being at work in full blast within a week after his arrival. Six or eight courses at a time will give even the most enthusiastic worker all he can attend to, and leave scant time for clinics, which occur at all hours of the day, but chiefly in the morning, the afternoon being largely given over to teaching.

The work in these courses usually consists of lectures and demonstrations upon the anatomy, physiology and pathology of the parts involved, operative work on the cadaver, and clinical demonstrations. Operative work on the living can also be gotten in some branches, but it is very expensive.

In diseases of the eye, ear, nose, and throat, there were, during the fall and winter of 1907-1908, almost as many students as in all other branches combined. Especially good is instruction in all branches of diseases of the eye, except refraction; not many Americans attempt to refract in Vienna. In ophthalmoscopy, in twenty lessons, the student, after a few lectures on diseases of the fundus, examines and is quizzed upon a hundred and fifty patients with pathological fundi—a most instructive course, which has its equal nowhere else in the world, save probably in Morefield Eye Hospital in London. In external diseases of the eye a dozen different cases are examined each day by each member of the class, after which he is closely quizzed upon the same. Little attention is paid to treatment; diagnosis and pathology being considered paramount. Other thorough courses are given on the bony structure of the orbit, anatomy of the eye, pathology, muscles, and operations on the cadaver and pigs' eyes.

In diseases of the ear, instruction is given on surgical diagnosis and treatment, anatomy and pathology, clinical work, operations on the cadaver, anatomy, physiology and pathology of the labyrinth, with special attention to the semicircular canals and nystagmus. The recent researches of Alexander and Barany upon mystagmus in connection with diseases of the labyrinth have aroused a great deal of interest in otological circles in Vienna, and the courses of each of these two scientific investigators are very popular with American students.

Diseases of the nose and throat are treated in the same thorough way, from anatomy up, and good operative courses on the living are offered. A very interesting course is that upon Bronchoscopy and Aesophagoscopy on the living, a branch which is receiving special attention from German and Austrian rhinologists at present.

In general medicine, good courses can be obtained in all branches, those in anatomy, pathology, bacteriology, internal medicine, pediatrics, skin, gynecology and obstetrics, being especially patronized by Americans.

Of the one hundred and ninety-five courses—many of which are duplicates by different professors—offered in the pamphlet published by the American Association of Vienna, eighty-four, or forty-three per cent., are upon diseases of the eye, ear, nose, and throat, and so great has been the demand for this work by Americans recently, that in many of these courses the fees have been materially increased.

The fees for English courses in Vienna run from four to twenty dollars, with an average of about ten dollars for a course of twenty hours, in all branches except operative courses on the living, which cost from four to ten times as much as the others. If one understands German well he can obtain many University courses free, and others at a nominal cost of from fifty cents to four dollars.

In London, work covering a period of two or three months costs from fifteen to forty-five dollars, and at Moorefield Eye Hospital the fee for six months' work as Junior Assistant is fifty dollars, which covers every course offered by the institution, and further confers a life ticket of admission to the hospital clinics.

The student may count upon investing an average of fifty dollars a month for tuition in Berlin and Vienna, and about one-half that amount in London.

Besides London, Berlin, and Vienna, there are numerous other cities which offer facilities for post-graduate work, such as Edinburgh, and, in Germany, Heidelberg, Halle, Leipzig, Freidburg, etc., but in the latter cities a speaking knowledge of German is imperative. In Halle, very thorough and practical work on the ear can be gotten, including several mastoid operations on the living, for one hundred dollars.

The Physician contemplating a trip abroad for study, frequently asks which season of the year is best for his undertaking, and how long he must remain abroad to accomplish his work. To the former question the answer is that fall or late summer is generally the best time to set forth, because the fall and winter months are the busiest in European Hospitals, there are more Americans abroad then, ensuring the rapid filling of all courses, and the tide of transatlantic travel has by that time turned westward, causing low rates and abundant room for the Eastbound traveler. Work can be obtained throughout the summer months, but many of the chief surgeons are off duty at that time, and their clinics are conducted by first assistants. The Christmas holidays interfere but little with courses, the majority of them suspending from one to several days.

As to the length of the stay, this depends upon various considerations; the length of tim which the busy physician can concientiously spend away from home and practice, his inclinations, the condition of his pocket-book, etc.; but, generally speaking, he should allow two weeks for travelling each way, a week or ten days lost getting to work, and four weeks for each set of courses, if he goes to Germany or Austria. A somewhat shorter time for travelling should be allowed if he goes to London only, but from two to five months for study in the latter city, the instruction not being divided into so many and such short courses as on the continent.

The experienced man who merely wishes to brush up on a few subjects, or do general work, can accomplish a great deal in an absence of three months, one month of which is spent going and coming, and two at work, though he can find enough to keep him busy for an unlimited period. A six month's absence is much more desirable if it can possibly be managed, the practice at home will not suffer in the long run, and one can accumulate a marvellous amount of experience and knowledge in that length of time. Those who spend as much as two years abroad are to be envied, as well for their great store of experience, as for the thorough, careful and scientific methods which they are taught to pursue.

It is doubtful if the freshly graduated student of medicine should proceed immediately to Europe for post-graduate work. He does not realize how little he really knows, and it takes a few years in general practice, or a year or so in hospital work to show him wherein he is deficient, and along what lines he should apply his energy. Then let him go to Europe if he wishes, and he will get the maximum benefit of such a trip.

Living expenses are moderate, compared with corresponding expenses in our own large cities. The average price paid for board and room by the American student abroad is thirty-five dollars a month, though some pay less and some a good deal more. Cheaper board can always be obtained at a greater distance from the hospital district, but it is not advisable to take it on account of the valuable time consumed coming and going, and because one will not be thrown with American companions. In London nearly all Amricans board along the several streets leading out of Russell Square, as this locality is situated in the center of the hospital district and Metropolitan London. In Vienna they frequent the side streets close to the "Allgemeine Krankenhaus," and in Berlin they are scattered about the city on account of the widely separated hospitals.

The student can easily live upon forty-five dollars a month anywhere in England, Germany or Austria, which sum includes every expense except for traveling and for tuition. Travelling expenses from Georgia to Europe and return, via New York, will be from two hundred and twenty-five to three hundred dollars, according to route chosen, number of cities stopped in, etc. This includes every expense en route; tickets, sight-seeing, hotel bills, tips, cabs, etc., in eight or ten cities, first-class fare on all boats, and second class on railways. A doctor starting from some southern state can, during an absence of four months, get three months of post-graduate work in any European medical center, and pay all expenses, with five hundred dollars. For every month beyond four, a hundred dollars may be added.

A few practical points for the benefit of the inexperienced traveller abroad will not be out of place here. It is best to carry any sum, under a thousand dollars, in the form ot American Express checks, as these are accepted everywhere without further identification than the signature, but larger sums should be invested in a letter of credit. For a stay of six months or less, do not carry a trunk, as only hand luggage is transported free on the continent, baggage rates are very high, and customs examinations are tedious and troublesome when there is a trunk to look up and open at every frontier; but rather carry two large suit cases as hand luggage, and turn them over to a porter whenever they must be moved, for which service the usual tip is the equivalent of five or ten cents, according to circumstances. Second class compartments are good enough for all save fools, nobles and millionaires, on continental railways, and even third-class is tolerable in England.

The sea route, shortest in distance, is from New York to Plymouth, and that shortest in time, from New York to Liverpool by the new monster turbiners of the Cunard Line. Berlin is best reached by boat from New York to Hamburg, and thence a short trip by rail, or via London and a twenty hour run by the Hook of Holland route. Vienna can be reached either across the continent a thirty-six hour journey from London, or thirty hours from the port of Cherbourg, France, through Paris and Switzerland, or through the Mediterranean to Naples or Genoa, across

Italy and Southwestern Austria. The trip through the Mediterranean to Naples, then through Rome, Florence, Venice, the beautiful Austrian Alps and Semmering Pass, to Vienna, is very beautiful and interesting, but to those who suffer the horrors of mal de mer it has a drawback in the twelve or fourteen day ocean passage.

The railways of Europe will sell the traveller cheap "rundreise" tickets, good for as long as ninety days, over any route selected by himself, no matter how devious, and allow stopovers in any cities along the way. Thus there are many interesting and instructive itineraries from which to choose, and by investing a little more money and a few days' time, many historical cities can be visited, even though superficially, and the mind of the disciple of Aesculapius broadened by this pardonable digression from the path of duty.

---

After many years' work a modern medical library is almost ready for the use of physicians of Nashville and Davidson county. The library will have about 3,000 volumes and many medical journals will be kept on file. The library will be kept and cared for as a separate part of Carnegie Library. Should not the physicians of Atlanta carry forward her plan for a similar library?

---

The following changes are announced in the University College of Medicine, Richmond: Professor of theoretical pediatrics, Dr. McGuire Newton; clinical pediatrics, Dr. Paulus A. Irving; theiry and practice of medicine, Dr. Alexander G. Brown, Jr.; and practice of medicine and physical diagnosis, Dr. J. Garnett Nelson.

---

The appointment of night inspectors in tenements to prevent the overcrowding of sleeping rooms is urged by representatives of the Federated Charities of Baltimore. Italian laborers are said to be the chief offenders, and one instance is reported where nearly a score occupied a room intended for two persons. The city authorities approve the suggestion.

